# Implementing Peer Learning in Clinical Nursing Education: Addressing Challenges in High-Pressure Healthcare Systems—An Interview Study

**DOI:** 10.1177/23779608251399270

**Published:** 2025-11-20

**Authors:** Ann-Sofie Källberg, Marie Elf, Ulrika Förberg, Ulrika Nelzén Sievertsson, Henrietta Forsman, Maria Svedbo-Engström

**Affiliations:** 1 Caring Science, 101092 Dalarna University School of Health and Welfare, Falun, Sweden; 2 Centre for Clinical Research Dalarna, Falun, Sweden; 3 Center for Healthcare Education, 3318 Region Dalarna, Falun, Sweden; 4 Department of Health and Welfare, Region Dalarna, Falun, Sweden

**Keywords:** nursing students, nursing education, qualitative research

## Abstract

**Introduction:**

Peer learning is an educational strategy used in clinical training for nursing students that has proven to be beneficial for both students and supervisors.

**Objective:**

This study aimed to describe the perceptions and experiences of first-line managers, clinical supervisors, and educators in nursing with peer learning (PL) as a supervision model. It also aimed to describe the implementation process, focusing on key factors and obstacles to successful implementation in healthcare systems facing pressure from registered nurse (RN) shortages.

**Method:**

This qualitative descriptive interview study involved individual and focus group interviews with 20 participants from three groups: first-line managers, clinical supervisors, and educators in nursing. Interviews were analyzed using deductive content analysis, guided by the i-PARIHS framework.

**Results:**

While PL improved supervision quality, adaptability, and recruitment opportunities, it required active engagement from staff and students, and ongoing collaboration with the university. Experienced challenges were students with varying competence levels and inadequate continuity due to the shortage of RNs. Participants found the introductory activities and scheduled meetings valuable but faced challenges related to time allocation and a lack of RNs to act as clinical supervisors.

**Conclusion:**

Despite PL's benefits, the RN shortage hinders its implementation, necessitating the development of flexible models that can be implemented despite the shortage.

## Introduction

Clinical training is fundamental in nursing education, shaping students’ competence and preparedness for professional practice. Peer-learning (PL) is a supervision model, and research shows PL significantly benefits nursing students by increasing self-confidence and contributes to their professional development, teamwork, and communication skills (Markowski et al., 2021; Nelwati et al., 2018; Pålsson et al., 2017). In Sweden, a bachelor's nursing program requires students to complete 50% of their education in a clinical setting. In response to the shortage of RNs, the Swedish government has mandated an increase of nursing students, leading to a higher demand for clinical education (Swedish Government Official Support (SOU), 2024). At the same time, the entire Swedish healthcare system is under pressure due to a lack of registered nurses (RNs) (National Board of Health and Welfare, 2022; Selberg & Mulinari, 2022). This has also been a global issue before and after the COVID-19 pandemic (Buchan & Aiken, 2008; International Council of Nurses, 2021). Moreover, the COVID-19 pandemic has significantly transformed nursing education, shifting it from traditional classroom-based learning to predominantly online and hybrid formats (Tang et al., 2022). Healthcare organizations must provide high-quality clinical training, ensure student satisfaction (Lee et al., 2021; Rodríguez-García et al., 2021), and support the recruitment of competent RNs, ultimately securing the overall quality of the healthcare system (National Board of Health and Welfare, 2018).

## Review of Literature

PL involves students working together, sharing experiences, discussing cases, and solving problems as a team in real-life patient care situations (Boud et al., 2013). It benefits clinical supervisors by fostering independence among students and reducing their workload (Nygren & Carlson, 2017; Olsson et al., 2020). Further, PL contributes to a supportive learning environment in clinical settings (Dyar et al., 2024, 2021). However, supervising two students for effective PL can be stressful. Thus, implementing PL requires a careful selection of student pairs, support for clinical supervisors, and comprehensive knowledge of PL (Henderson et al., 2020; Nygren & Carlson, 2017).

It is essential to have a well-supported implementation strategy to successfully introduce new methods. In the context of healthcare, Promoting Action on Research Implementation in Health Services (PARIHS) (Rycroft-Malone, 2004) is a frequently used framework that has been further developed into the integrated-PARIHS (i-PARIHS) framework (Harvey & Kitson, 2016). The i-PARIHS framework emphasizes *facilitation* (“a technique by which one person makes things easier for others,” according to Kitson et al., 1998, p. 152) as the active ingredient in the implementation process, which is focused on three factors: the *innovation* to be implemented (e.g., how it fits with previous working methods and its usability), the *recipient* (individual and collective) and the *context*. Leadership at local and organizational levels is vital for supporting and enabling change, and *context* includes leadership as an important factor. Facilitation can be internal or external (Harvey et al., 2002; Kitson et al., 1998).

Most previous studies on PL in healthcare have focused on the perspectives of students and clinical supervisors (Considine et al., 2021; Henderson et al., 2020; Nelwati et al., 2018; Nygren & Carlson, 2017; Pålsson et al., 2022; Stone et al., 2013). There is limited research on the implementation process, including the experiences of managers, clinical supervisors, and educators in nursing. Additionally, there is a gap in understanding key factors influencing the introduction and implementation of PL in healthcare systems facing pressure from RN shortages.

## Objectives

- To describe the perceptions and experiences of first-line managers, clinical supervisors, and educators in nursing with the PL model during and after its introduction.
- To describe the implementation process, focusing on key factors and obstacles to successful implementation in a high-pressure work environment.

## Methods

### Design

This study used a descriptive qualitative design, analyzing semistructured individual interviews and focus groups to gain insight into the participants’ perceptions and experiences. The data was analyzed using deductive content analysis, with the predefined factors of the i-PARIHS framework as guiding themes.

### Sample and Setting

This study is part of a pilot project implementing PL as a clinical education model in a bachelor's nursing program, conducted by a healthcare region and a university in Sweden. The PL project involved the supervision of a selected group of students within the bachelor's nursing program who completed their clinical education in the fall semester of 2017 and spring semester of 2018. The project was led by a group of two researchers, two project managers representing the healthcare region, and five representatives from the university's bachelor's nursing program. The participants from the healthcare region in the project group varied over time.

Before the start of the project, the project managers used e-mail to contact four managers of healthcare organizations (representing primary care, psychiatric care, surgical care, and internal medicine) and inform them about the project. These managers notified interested first-line managers who received further information and discussed potential involvement. Using purposive sampling for heterogeneous representation, 11 departments agreed to participate in the pilot project.

The study involved departments from three hospitals and one primary healthcare center, including psychiatric, surgical, internal medicine, orthopedic, infectious disease, and palliative care units. For the interview study, the researchers selected participants directly affiliated with these units. Participants were eligible for inclusion if they held an active role in the project. First-line managers, internal facilitators (i.e., clinical supervisors), and educators from the university's bachelor's nursing program were invited via e-mail, which included details about the study's purpose and the participants’ rights. A total of 20 female participants were interviewed: eight first-line managers, seven internal facilitators representing all participating departments, and five educators in nursing. Educators in nursing are RNs with master's degrees who teach and prepare nursing students for clinical practice, working in academia, and clinical settings.

### Procedure of the Pilot Project

**Figure 1.**
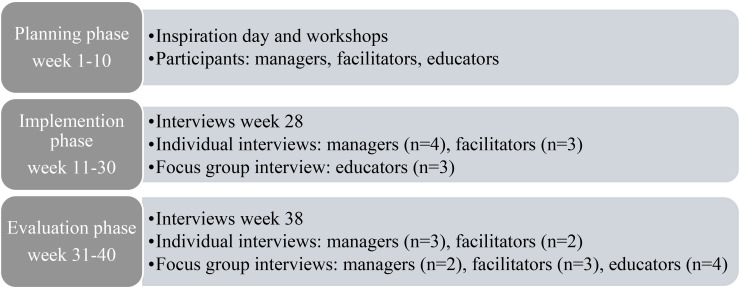
The planning, implementation, and evaluation phases and data collection were performed during autumn 2017 and spring 2018.

The pilot project began in the fall of 2017 with a 10-week planning phase, followed by a 20-week implementation phase and a 10-week evaluation phase (Figure 1). Guided by the i-PARIHS framework, both internal and external facilitators were recruited. The internal facilitator was a key person at the participating departments. An inspiration day was organized for the regional departments and nursing educators covering lectures and discussions on PL. In addition, a 1-day workshop was held for the first-line managers, the internal facilitators, and the educators. This workshop introduced PL and included training in designing structured learning activities. Throughout the project, the internal facilitators were offered monthly meetings to discuss and share their experiences of PL and the implementation process.

### Data Collection

Those who agreed to participate in the interviews were contacted by the project group to schedule a time, location, and mode of their choosing for the interview. Individual telephone interviews were mostly chosen for practical reasons (e.g., long distance) to increase the opportunity to participate. The focus groups interviews were conducted face-to-face on site. The focus groups were homogeneous (i.e., participants with the same role in the project were placed in the same focus group) to enable the participants to share different or common experiences with the PL and its implementation. The interviews were carried out on two different occasions to study the participants’ experiences with the PL model and the implementation process over time. The first interviews were conducted at the end of the implementation phase, and the second interviews were conducted 10 weeks after the first interviews, during the evaluation phase. One first-line manager, one internal facilitator, and four educators were interviewed both times (Figure 1).

The interviews were conducted by members of the project group and by a third researcher with experience in and knowledge about the healthcare sector, implementation science, and PL. The individual interviews lasted 10 to 45 min, and the focus group interviews lasted 20 to 47 min. All interviews were recorded with a digital voice recorder and transcribed verbatim. The semistructured interview guide used in both the individual interviews and the focus groups was developed by the researchers, based on the structure of the i-PARIHS framework—that is, facilitation, innovation, recipient, and context. The interviews began with a question about the participants’ experience of their role in the project, followed by their experience with the arrangement and implementation process of the PL. Subsequent questions focused on the context, such as the support, conditions, time, and resources the participants had received or had been missing. Further questions inquired about the anchoring of the PL, such as any obstacles, difficulties, challenges, and success factors; finally, the participants were asked if there was anything else they wanted to add. No new information emerged during the final interviews.

### Research Ethics

The study was approved by a Research Ethics Review Board and managers in the healthcare region. Participants received written information before the interviews and could withdraw at any time without explanation. Participation in the interviews was consent. Results are reported at the group level to protect identities.

### Analysis

The researchers used deductive content analysis (Elo & Kyngäs, 2008) using the i-PARIHS framework as a matrix (comprising the *innovation*, the *recipients*, and the *context*). The textual data were manually arranged and sorted using an Excel spreadsheet. The researchers analyzed the data separately for the first-line managers, internal facilitators, and educators with the intention to identify similarities or differences between the groups. First, the researchers read each interview in its entirety during the preparation phase to understand the whole; then, the researchers read the interviews a second time while focusing on text related to the predefined themes in the matrix. Next, the researchers extracted text related to the predefined themes and sorted it into one of the themes. The researchers created categories and subcategories related to the framework to provide a more detailed description. To ensure accuracy, a second researcher reviewed the extracted text from six interviews (two from each group) to confirm that the extracted texts were related to the predefined themes. In addition, one researcher and one healthcare professional who had conducted interviews reviewed the extracted text sorted into categories to reach a consensus.

## Results

The researchers present the result in three tables representing three themes for a successful implementation and challenges as per the i-PARIHS framework, along with the main categories and subcategories created from the interviews (Tables 1–3). The tables include the participants’ experience of factors that either contributed to or hindered a successful implementation.

**Table 1.** The Theme of Innovation and Its Identified Categories and Subcategories for **^1^** First-Line Managers, **^2^** Internal Facilitators, and **^3^** Educators.

Theme	*Innovation: Peer Learning*			
*Categories*	*Valuable Planning Phase*	*Enhanced Supervision Quality*	*A Flexible and Favorable Model*	*Addressing Challenges*
*Subcategories*	**^1^** *The planning phase was pedagogical and structured with inspiring activities* **^1^** *Regular meetings provide a valuable platform for exchanging experiences and reflections* **^2^** *The inspiration day and workshop proved to be immensely rewarding* **^2^** *Regular meetings with fellow facilitators proved to be advantageous* **^3^** *The planning phase was valuable*	**^1^** *The PL improved the structure and quality of the supervision* **^2^** *PL has been educational and developmental for both us and the students* **^2^** *PL might enhance satisfaction levels among both students and RNs* **^2^** *Reflection serves the process of development*	**^1^** *The PL model is flexible and can be locally adapted* **^2^** *The model offers flexibility for local adaption* **^1^** *PL could potentially enhance recruitment by offering an appealing supervision model* **^2^** *PL presents a valuable recruitment opportunity for RNs* **^2^** *There has not been a significant increase in workload* **^2^** *This project was not markedly different from previous experiences*	**^1^** *It is challenging to ensure that all students have comparable levels of knowledge and engagement* **^1^** *It is a challenge to continually assess the quantity and distribution of students in order to preserve the efficacy of the PL environment* **^2^** *Supervising students who were at varying levels of skills was challenging* **^3^** *Clear criteria are essential for effective utilization of the model* **^3^** *Addressing the challenge of students’ varying skill levels is crucial*

**Table 2.** The Theme of *Recipients* and Its Identified Categories and Subcategories for **^1^** First-Line Managers, **^2^** Internal Facilitators, and **^3^** Educators in Nursing.

Theme	Recipients		
Categories	*Resource Allocation Challenges*	*Turning Resistance Into Commitment and Development Ideas*	*Facilitator's Role and Support Needs*
*Subcategories*	**^1^** *Engaging in using the PL model is advantageous* **^1^** *There is a lack of resources, especially RNs* **^3^** *There is a lack of continuity because of staff turnover* **^3^** *The participation of first-line managers is important to allocate resources*	**^1^** *Expect initial resistance when introducing new methods* **^1^** *New ideas involve collaboration with other professionals* **^3^** *Initially, there was some resistance from supervisors* **^2^** *Colleagues and managers have mainly responded positively* **^2^** *Positive and engaged students are crucial*	**^3^** *Facilitators are involved in establishing routines and creating structured learning activities* **^3^** *Information, support and training are essential for all staff* **^3^** *Several educators should have been involved for support*

PL=peer learning; RN=registered nurse.

**Table 3.** The Theme of *Context* and Its Identified Categories and Subcategories for **^1^** First-Line Managers, **^2^** Internal Facilitators, and **^3^** Educators.

Theme	Context		
Categories	*Effective Communication and Support are Necessary*	*There are Challenges In the Implementation and Sustaining Change*	*Different Resources are Needed*
*Subcategories*	**^1^** *Clear and transparent communication is essential* **^2^** *Reaching out with information has been difficult* **^1^** *Local support for all staff and students is needed* **^3^** *Varied levels of support are needed*	**^1^** *It is necessary to prepare for successful project implementation* **^1^** *Engaged staff play a crucial role in making the project possible* **^1^** *Continuous feedback from the university is important* **^3^** *Better collaboration between the university and departments is needed* **^2^** *Maintaining the change is dependent on supervisors, which is very uncertain* **^3^** *The model must be tailored to the specific conditions*	**^2^** *More time is needed for preparation and organization* **^2^** *Several professionals should have been involved* **^2^** *The time and conditions for the assignment varied* **^3^** *The workload was increased without more time allocation* **^3^** *Active participation in creating structured learning activities is necessary*

### The Innovation

The first theme, *Innovation* focuses on the PL model's perceived benefits and challenges as articulated by the participants. PL was perceived to have a *valuable planning phase*, to lead to *enhanced supervision quality* and to be *flexible and favorable*. However, there were *also some challenges to address* (Table 1).

#### Valuable Planning Phase

With its structured and inspiring activities, the planning phase was highly valued by all the participants. First-line managers appreciated the pedagogical structure and the valuable regular meetings, which provided a platform for exchanging experiences. One internal facilitator (if) expressed: *“The arrangement was structured, and the activities and workshop were well-organised” (if 105).* The internal facilitators also valued collaborating with other facilitators, as it provided opportunities to share both problems and successes.

#### Enhanced Supervision Quality

PL was perceived to increase satisfaction for students and supervisors, better educational structure to improve supervision quality. Internal facilitators perceived PL to contribute to professional development and educational improvement. In a focus group discussion (fg), one internal facilitator expressed*: “I think PL can give a boost to the entire department, you learn a lot yourself too, it feels like we offer better quality” (fg 111).*

#### A Flexible and Favorable Model

First-line managers and internal facilitators found the PL flexible, allowing for local adaptation without increasing workload. It was viewed as a way to enhance recruitment opportunities for new RNs by offering an appealing supervision model.

#### Challenges to Address

The educators emphasized the need for supervisors to understand PL and have clear criteria for its use. One educator in nursing commented: “*If there is an intention to widely introduce PL, it would be interesting to know the framework. What is required for an organization to say that we practice PL?” (fg 1).* Supervising students with varying competence levels challenging and highlighted the need for students to be at the same competence level.

### The Recipients

The second theme, *Recipients*, highlights the perspective of those directly involved. As illustrated in Table 2, participants reported a range of experiences concerning *resource allocation challenges*, *turning resistance into commitments, and development ideas*, to the PL model as well as *facilitator's role and need for support*.

#### Resource Allocation Challenges

Challenges persisted due to the RN shortages, affecting student support continuity. Educators emphasized the importance of first-line managers’ active participation in allocating necessary time and resources. However, obstacles remained, particularly the availability of RNs. As one first-line manager (fl) put it: *“If PL is to survive, it is about the availability of RNs” (fl 112).*

#### Turning Resistance Into Commitment and Developmental Ideas

Initially, supervisors hesitated to oversee two students at the same time, but this concern diminished over time. Supervisors’ experiences evolved, collaboration increased, and new ideas emerged; in the end, PL was seen as beneficial. As one first-line manager stated, “*There was a concern before starting that this would take a lot of time, but that is not the case now” (fl 110).* Over time, the internal facilitators felt that colleagues and managers mostly experienced PL positively and expressed that positive and engaged students are crucial for the model to work.

#### Facilitator's Role and Support Needs

The internal facilitators were responsible for developing and adapting local routines and learning activities for their patient groups. The educators suggested more collaboration and support from internal facilitators would have been beneficial. They also emphasized the need for information, support, and training for all staff levels, not just RNs supervising nursing students (Table 2).

#### The Context

The third theme, *Context*, examines the broader organizational and environmental factors that influenced the implementation of the PL model. As detailed in Table 3, participants highlighted the necessity of *effective communication and support*, the *challenges of sustaining change*, and the need for *diverse resources*.

#### Effective Communication and Support are Necessary

The theme of context underscores the need for clear and transparent communication and the overall responsibility of first-line managers to provide information and support coworkers at both local and organizational levels. Internal facilitators faced challenges in disseminating information.

#### Challenges in the Implementation and Sustaining Change

First-line managers benefited from engaged staff and university collaboration. One first-line manager expressed: “*My coworkers are wise and engaged, I just need to be informed of how it is supposed to be carried out” (fl 110).* First-line managers and educators expressed that continuous collaboration between departments and the university was essential for successful and lasting implementation. One educator in nursing noted: “*Collaboration between the university and the healthcare organization requires ongoing meetings and information exchange. Simply introducing a model is insufficient; additional efforts are necessary” (en 1).*

Internal facilitators identified the shortage of RNs as a factor that impairs supervision prerequisites and the ability to maintain changes due to dependence on supervisors. However, they also described supervising students as the only way to ensure the future supply of RNs and considered that PL contributed to a good learning environment. One of the internal facilitators stated*: “This is indeed the only chance to secure the supply of RNs and to ensure a good practice environment where they want to come and stay with us” (if 111).* Educators highlighted the need for long-lasting commitment from the university and healthcare departments to ensure sustainability. An educator commented:

It's important that investments made by the university and the healthcare region into various initiatives yield sustainable results. It can be disheartening to see projects disappear after a lot of effort and resources have been put into them. It's crucial to ensure that these investments are not only beneficial in the short term but also have a long-lasting impact. *(fg 1)*

#### Different Resources are Needed

Some educators felt they lacked time to support supervisors due to their increased university workload. Balancing additional work within their limited time posed a challenge as did active participation in creating structured learning activities. They wanted more time to actively participate and address the varying needs of clinical departments. One educator commented: “*To different degrees, one unit resolved the issue independently, while the other extended an invitation to me” (fg 1).* Internal facilitators needed more time for preparation and organization and to involve multiple professionals. Educators experienced varied conditions during PL model implementation and emphasized the need for diverse support and better university–department collaboration (Table 3).

## Discussion

The results indicate that PL was perceived as beneficial for students and supervisors, in line with findings from previous studies (Jassim et al., 2022; Nygren & Carlson, 2017; Vuckovic & Landgren, 2021). The participants believed PL provided better structure and improved the quality of the supervision. Moreover, some participants suggested that PL could help to attract and retain RNs by offering a more structured and supportive learning experience. This is an important consideration during a time of increasing nursing workforce shortages in healthcare, which have significantly escalated during and after the COVID-19 pandemic (International Council of Nurses, 2021). However, the ongoing and significant shortage of RNs is at the same time a critical challenge to sustaining PL in healthcare as its implementation depends on resources that are limited (National Board of Health and Welfare, 2018; Rosenberg, 2019).

Participants valued the planning phase, particularly the introduction and educational activities. However, it was suggested that the inclusion of additional student-focused activities, such as preparatory workshops or follow-up discussions, could further increase PL compliance and engagement (Nelwati et al., 2018). First-line managers appreciated meetings with other departments, while internal facilitators valued connections with peers from other departments.

The use of internal and external facilitators aligned with the i-PARIHS model was considered beneficial. Despite its benefits, supervising students at different competence levels was challenging, as confirmed by previous studies (Henderson et al., 2020; Nygren & Carlson, 2017). This complexity demands tailored support strategies to ensure learning outcomes are met for all students.

Although the PL model was generally seen as positive, challenges arose from the recipients and context. First-line managers struggled with resource allocation, especially managing RNs, and felt responsible for the project's success while keeping staff engaged. This is consistent with theories on improvement work, which highlight the critical role of management and leadership in sustaining change (Granberg et al., 2021; Harvey & Kitson, 2016).

While internal facilitators emphasized the importance of working with positive colleagues and engaged students, they also expressed a need for more planning time and support from university educators, which is consistent with the results from other studies (Cusack et al., 2020; Henderson et al., 2020). Educators appeared to recognize these needs and desired more time to fulfill their roles. Moreover, collaboration with educational institutions seems to play a significant role in successful PL implementation, as supported by the Association of Swedish Higher Education Institutions (Swedish Government Official Support (SOU), 2024). However, despite positive views of PL, the lack of RNs remains a major obstacle in supervising new RNs, which continues to hinder progress in implementing PL.

Clinical training is unquestionably a fundamental role in nursing education, and the PL model has been recognized for its potential to ease pressures on RNs while enabling the admission of more students, which are greatly needed. However, a paradox emerges, while more RNs are urgently required, the shortage of RNs and limited clinical placements make it difficult to provide adequate supervision for students. This creates a kind of vicious cycle. Studies highlight the benefits of PL in addressing these challenges (Nygren & Carlson, 2017; Olsson et al., 2020), yet others suggest that its implementation alone is not sufficient to resolve the broader systemic issues (Rosenberg, 2019; Swedish Government Official Support (SOU), 2024). However, a study has shown that despite the continued shortage of RNs following the COVID-19 pandemic, PL can be used as a supervision model with existing staff, enabling students to achieve their learning goals (Al-Momani et al., 2025). This raises important questions about the extent to which PL can serve as a long-term solution, or whether additional structural changes are needed.

### Strengths and Limitations

The fewer-than-expected focus group participants may affect the depth of the findings. The focus groups were smaller than anticipated due to some participants’ absence, attributed to time constraints. Despite this, data saturation was achieved. The process of coding and categorization was primarily conducted by the first author, with extensive team discussions to ensure trustworthiness. The diverse professions and work locations of participants enhance the study's trustworthiness. The transferability of the findings to similar healthcare settings is plausible, as the findings align with other studies on PL. Internationally, many healthcare systems face challenges with RN shortages and clinical placements for students.

### Clinical Implications and Further Research

To maintain the quality and sustainability of PL, it is important to establish support structures, such as ongoing training for supervisors and mechanisms for knowledge transfer when staff turnover occurs. Research is needed to explore the long-term sustainability of PL, including how it can be maintained over time despite staff turnover and organizational changes.

## Conclusion

PL was perceived to lead to enhanced supervision quality and to be flexible and favorable. However, there were also some challenges to address. Despite PL's benefits, the RN shortage hinders its implementation, necessitating the development of flexible models that can be implemented despite the shortage.

## Supplemental Material

sj-docx-1-son-10.1177_23779608251399270 - Supplemental material for Implementing Peer Learning in Clinical Nursing Education: Addressing Challenges in High-Pressure Healthcare Systems—An Interview Study Supplemental material, sj-docx-1-son-10.1177_23779608251399270 for Implementing Peer Learning in Clinical Nursing Education: Addressing Challenges in High-Pressure Healthcare Systems—An Interview Study by Ann-Sofie Källberg, Marie Elf, Ulrika Förberg, Ulrika Nelzén Sievertsson, Henrietta Forsman and Maria Svedbo-Engström in SAGE Open Nursing

sj-docx-2-son-10.1177_23779608251399270 - Supplemental material for Implementing Peer Learning in Clinical Nursing Education: Addressing Challenges in High-Pressure Healthcare Systems—An Interview Study Supplemental material, sj-docx-2-son-10.1177_23779608251399270 for Implementing Peer Learning in Clinical Nursing Education: Addressing Challenges in High-Pressure Healthcare Systems—An Interview Study by Ann-Sofie Källberg, Marie Elf, Ulrika Förberg, Ulrika Nelzén Sievertsson, Henrietta Forsman and Maria Svedbo-Engström in SAGE Open Nursing

## References

[bibr1-23779608251399270] Al-Momani S. M. Najjar Y. W. Shawagfeh M. T. Bsool A. A. AL-Zayyat A. A. Hdaib M. D. Al-Khatib M. R. Da’seh A. (2025). Facilitating nursing students’ clinical education continuity utilizing collaborative critical friendship approach: A quasi-experimental study. International Journal of Nursing Education, 17(1). 10.37506/2xjszr58

[bibr2-23779608251399270] Boud D. Cohen R. Sampson J. (2013). Peer learning in higher education: learning from and with each other (2nd ed.). Routledge. 10.4324/9781315042565

[bibr3-23779608251399270] Buchan J. Aiken L. (2008). Solving nursing shortages: A common priority. Journal of Clinical Nursing, 17(24), 3262–3268. 10.1111/j.1365-2702.2008.02636.x 19146584 PMC2858425

[bibr4-23779608251399270] Considine J. Berry D. Allen J. Hewitt N. Oldland E. Sprogis S. K. Currey J. (2021). Team-based learning in nursing education: A scoping review. Journal of Clinical Nursing, 30(7–8), 903–917. 10.1111/jocn.15599 33331081

[bibr5-23779608251399270] Cusack L. Thornton K. Drioli-Phillips P. G. Cockburn T. Jones L. Whitehead M. Prior E. Alderman J. (2020). Are nurses recognised, prepared and supported to teach nursing students: Mixed methods study. Nurse Education Today, 90, 104434. 10.1016/j.nedt.2020.104434 32315837

[bibr6-23779608251399270] Dyar A. Henriksson P. Stenfors T. Lachmann H. Kiessling A. (2024). Differences in supervision on peer learning wards: A pilot survey of the supervisor's perspective. Advances in Medical Education and Practice, 15, 85–96. 10.2147/AMEP.S439968 38327849 PMC10849097

[bibr7-23779608251399270] Dyar A. Stenfors T. Lachmann H. Kiessling A. (2021). What about the supervisor? Clinical supervisors’ role in student nurses’ peer learning: A phenomenographic study. Medical Education, 55(6), 713–723. 10.1111/medu.14436 33325543 PMC8246972

[bibr8-23779608251399270] Elo S. Kyngäs H. (2008). The qualitative content analysis process. Journal of Advanced Nursing, 62(1), 107–115. 10.1111/j.1365-2648.2007.04569.x 18352969

[bibr9-23779608251399270] Granberg A. Matérne M. Lundqvist L. O. Duberg A. (2021). Navigating change—managers’ experience of implementation processes in disability health care: A qualitative study. BMC Health Service Research, 21(1), 571. 10.1186/s12913-021-06570-6 PMC8190840 34112151

[bibr10-23779608251399270] Harvey G. Kitson A. (2016). PARIHS revisited: From heuristic to integrated framework for the successful implementation of knowledge into practice. Implementation Science, 11(1), 33. 10.1186/s13012-016-0398-2 27013464 PMC4807546

[bibr11-23779608251399270] Harvey G. Loftus-Hills A. Rycroft-Malone J. Titchen A. Kitson A. McCormack B. Seers K. (2002). Getting evidence into practice: The role and function of facilitation. Journal of Advanced Nursing, 37(6), 577–588. 10.1046/j.1365-2648.2002.02126.x 11879422

[bibr12-23779608251399270] Henderson S. Needham J. van de Mortel T. (2020). Clinical facilitators’ experience of near peer learning in Australian undergraduate nursing students: A qualitative study. Nurse Education Today, 95, 104602. 10.1016/j.nedt.2020.104602 33002746

[bibr13-23779608251399270] International Council of Nurses. (2021). *Policy brief: The global nursing shortage and nurse retention*. https://www.icn.ch/node/1297

[bibr14-23779608251399270] Jassim T. Carlson E. Bengtsson M. (2022). Preceptors’ and nursing students’ experiences of using peer learning in primary healthcare settings: A qualitative study. BMC Nursing, 21(1), 66. 10.1186/s12912-022-00844-y 35313874 PMC8939121

[bibr15-23779608251399270] Kitson A. Harvey G. McCormack B. (1998). Enabling the implementation of evidence based practice: A conceptual framework. Quality and Safety in Health Care, 7(3), 149–158. 10.1136/qshc.7.3.149 PMC2483604 10185141

[bibr16-23779608251399270] Lee M. Na H. M. Kim B. Kim S. Y. Park J. Choi J. Y. (2021). Mediating effects of achievement emotions between peer support and learning satisfaction in graduate nursing students. Nurse Education in Practice, 52, 103003. 10.1016/j.nepr.2021.103003 33774568

[bibr17-23779608251399270] Markowski M. Bower H. Essex R. Yearley C. (2021). Peer learning and collaborative placement models in health care: A systematic review and qualitative synthesis of the literature. Journal of Clinical Nursing, 30(11–12), 1519–1541. 10.1111/jocn.15661 33461240

[bibr18-23779608251399270] National Board of Health and Welfare. (2018). *Provision of competency and patient safety: How shortfalls in staffing and competence affect patient safety*. https://www.socialstyrelsen.se/publikationer/kompetensforsorjning-och-patientsakerhet–hur-brister-i-bemanning-och-kompetens-paverkar-patientsakerheten-2018-2-15/

[bibr19-23779608251399270] National Board of Health and Welfare. (2022). *Assessment of supply and demand for licensed personnel in healthcare and dental care—National planning support*. https://www.socialstyrelsen.se/publikationer/bedomning-av-tillgang-och-efterfragan-pa-legitimerad-personal-i-halso–och-sjukvard-samt-tandvard–nationella-planeringsstodet-2022-2022-2-7759/

[bibr20-23779608251399270] Nelwati, Abdullah K. L. Chan C. M. (2018). A systematic review of qualitative studies exploring peer learning experiences of undergraduate nursing students. Nurse Education Today, 71, 185–192. 10.1016/j.nedt.2018.09.018 30293048

[bibr21-23779608251399270] Nygren F. Carlson E. (2017). Preceptors’ conceptions of a peer learning model: A phenomenographic study. Nurse Education Today, 49, 12–16. 10.1016/j.nedt.2016.10.015 27865125

[bibr22-23779608251399270] Olsson C. Carlson E. Sundin-Andersson C. Josse-Eklund A. (2020). All our problems solved? Implementing peer learning in a geriatric hospital setting: A discussion paper. Nordic Journal of Nursing Research, 41(2), 61–64. 10.1177/2057158520975307

[bibr23-23779608251399270] Pålsson Y. Engström M. Swenne C. L. Mårtensson G. (2022). A peer learning intervention in workplace introduction—managers’ and new graduates’ perspectives. BMC Nursing, 21(1), 12. 10.1186/s12912-021-00791-0 34983518 PMC8725265

[bibr24-23779608251399270] Pålsson Y. Mårtensson G. Swenne C. L. Adel E. Engström M. (2017). A peer learning intervention for nursing students in clinical practice education: A quasi-experimental study. Nurse Education Today, 51, 81–87. 10.1016/j.nedt.2017.01.011 28142097

[bibr25-23779608251399270] Rodríguez-García M. C. Gutiérrez-Puertas L. Granados-Gámez G. Aguilera-Manrique G. Márquez-Hernández V. V. (2021). The connection of the clinical learning environment and supervision of nursing students with student satisfaction and future intention to work in clinical placement hospitals. Journal of Clinical Nursing, 30(7–8), 986–994. 10.1111/jocn.15642 33432645

[bibr26-23779608251399270] Rosenberg K. (2019). RN Shortages negatively impact patient safety. The American Journal of Nursing, 119(3), 51. 10.1097/01.Naj.0000554040.98991.23 30801322

[bibr27-23779608251399270] Rycroft-Malone J. (2004). The PARIHS framework—a framework for guiding the implementation of evidence-based practice. Journal of Nursing Care Quality, 19(4), 297–304. 10.1097/00001786-200410000-00002 15535533

[bibr28-23779608251399270] Selberg R. Mulinari P. (2022). Exit spirals in hospital clinics: Conceptualizing turnover contagion among nursing staff. Scandinavian Journal of Public Administration, 26(1), 87–107. 10.58235/sjpa.v26i1.7045

[bibr29-23779608251399270] Stone R. Cooper S. Cant R. (2013). The value of peer learning in undergraduate nursing education: A systematic review. ISRN Nursing, 2013, 930901. 10.1155/2013/930901 23691355 PMC3649279

[bibr30-23779608251399270] Swedish Government Official Support (SOU). (2024). *Enhanced collaboration for clinical training—Long-term measures for nursing programmes*. https://www.regeringen.se/rattsliga-dokument/statens-offentliga-utredningar/2024/02/sou-20249/

[bibr31-23779608251399270] Tang Y. M. Lau Y.-Y. Chau K. Y. (2022). Towards a sustainable online peer learning model based on student's perspectives. Education and Information Technologies, 27(9), 12449–12468. 10.1007/s10639-022-11136-y 35668899 PMC9157034

[bibr32-23779608251399270] Vuckovic V. Landgren K. (2021). Peer learning in clinical placements in psychiatry for undergraduate nursing students: Preceptors and students’ perspective. Nursing Open, 8(1), 54–62. 10.1002/nop2.602 33318812 PMC7729660

