# Supplementary material for: Implementing Peer Learning in Clinical Nursing Education: Addressing Challenges in High-Pressure Healthcare Systems—An Interview Study
Source: SAGE Open Nurs. 2025 Nov 20;11:23779608251399270. doi: 10.1177/23779608251399270 (PMC12638713; doi:10.1177/23779608251399270)
Supplement: sj-docx-1-son-10.1177_23779608251399270 - Supplemental material for Implementing Peer Learning in Clinical Nursing Education: Addressing Challenges in High-Pressure Healthcare Systems—An Interview Study [file sj-docx-1-son-10.1177_23779608251399270.docx]

**Interview Guide for Educators in Nursing/ First-line managers/ Internal facilitators**

**• The role as an Educator in Nursing/ First-line manager/ Internal facilitator**
Can you describe what your role has been in the implementation of Peer Learning so far?
For example, what have you concretely done, activities, etc.?
Who have you collaborated with in your role (students, representatives in clinical organizations, etc.)? How has it worked? What significance has it had?

**• Structure of the pilot project**
How have you perceived the activities (meeting forums, workshops, etc.) that have been offered? What significance have these had for you in your role?

**• Support, conditions, and resources**
What type of support, conditions, and resources have you received? Has it been sufficient? If not, what would have been needed?

**• Time**
How much time have you spent working on the implementation of Peer Learning? Has the time felt sufficient? If not, how much time would you have needed and for what?

**• Obstacles, difficulties, challenges**
Have you encountered any such issues related to the implementation of Peer Learning? Which ones? What have you been able to do to address these?

**• Success factors**
Are there things that you feel have gone particularly smoothly and worked especially well? What? Why?

**• Other**
Is there anything else related to the implementation of Peer Learning that we have not already discussed but that you would like to highlight?

**Additional for data collection 4**
We are also interested in knowing how you view the continued application of the PL model in the organizations that have now participated in the pilot project.

**• Sustainability of the model in the organization**
Have you perceived that there are plans for how the Peer Learning model will be continued in the organizations you are in contact with? If so, how do you perceive that this has been planned (organization, structure, etc.)?
Is there a need for support, conditions, and resources for you in your role as an educator in nursing/ first-line manager/ internal facilitator in order for you to continue supporting the sustainability of the Peer Learning model in clinical organizations? In what form? From whom can you obtain this?
